# Survival and comorbidities in lung cancer patients: Evidence from administrative claims data in Germany

**DOI:** 10.32604/or.2022.027262

**Published:** 2023-01-31

**Authors:** DIEGO HERNANDEZ, CHIH-YUAN CHENG, KARLA HERNANDEZ-VILLAFUERTE, MICHAEL SCHLANDER

**Affiliations:** 1 Division of Health Economics, German Cancer Research Center (DKFZ), Heidelberg, Germany; 2 Medical Faculty Mannheim, Heidelberg University, Mannheim, Germany

**Keywords:** Lung Cancer, Comorbidities, Survival, Administrative data, Statutory health insurance

## Abstract

Lung cancer is the most common cancer type worldwide and has the highest and second highest mortality rate for men and women respectively in Germany. Yet, the role of comorbid illnesses in lung cancer patient prognosis is still debated. We analyzed administrative claims data from one of the largest statutory health insurance (SHI) funds in Germany, covering close to 9 million people (11% of the national population); observation period was from 2005 to 2019. Lung cancer patients and their concomitant diseases were identified by ICD-10-GM codes. Comorbidities were classified according to the Charlson Comorbidity Index (CCI). Incidence, comorbidity prevalence and survival are estimated considering sex, age at diagnosis, and place of residence. Kaplan Meier curves with 95% confidence intervals were built in relation to common comorbidities. We identified 70,698 lung cancer incident cases in the sample. Incidence and survival figures are comparable to official statistics in Germany. Most prevalent comorbidities are chronic obstructive pulmonary disease (COPD) (36.7%), followed by peripheral vascular disease (PVD) (18.7%), diabetes without chronic complications (17.4%), congestive heart failure (CHF) (16.5%) and renal disease (14.7%). Relative to overall survival, lung cancer patients with CHF, cerebrovascular disease (CEVD) and renal disease are associated with largest drops in survival probabilities (9% or higher), while those with PVD and diabetes without chronic complications with moderate drops (7% or lower). The study showed a negative association between survival and most common comorbidities among lung cancer patients, based on a large sample for Germany. Further research needs to explore the individual effect of comorbidities disentangled from that of other patient characteristics such as cancer stage and histology.

## Introduction

Lung cancer is the most common cancer type worldwide with close to 2.1 million incident cases in year 2019 [1]. In Germany, 59,221 individuals were newly diagnosed with lung cancer and 44,881 died of it in year 2019 [2]. It is the leading and second leading cancer type in terms of mortality for men and women respectively, causing 22% of cancer related deaths in men and 16% in women [2]. Incidence is expected to increase in the following years, mainly driven by the diagnoses for women [3].

There is a consensus that the comorbidity burden among lung cancer patients is high [4–7]. This is because of the strong relationship between smoking and lung cancer, cardiovascular diseases and other respiratory tract diseases. Cardiac toxicity following radiotherapy, chemotherapy or immunotherapy may contribute to the risk of concomitant diseases after treatment as well [8]. Comparative worse prognoses for lung cancer patients with comorbidities is mainly a result of lower odds of underdoing surgical resection, receiving or completing chemotherapy treatment, as well as therapy dose reduction, increasing the likelihood of an extended length of stay in hospital and of developing postoperative complications [9–17]. The presence of concomitant diseases could also interfere with a complete diagnostic evaluation and consequently accurate staging. Comorbidities might also be related to shorter survival because of their association with older ages [16].

Nevertheless, researchers have not yet fully agreed on the impact comorbidities might have on lung cancer patient chances of survival. Some studies have indicated a no significant to small effect [6,18–21]. These studies observed lung cancer patients with comorbidities had a similar prognosis to those without comorbidities. It has been suggested that the absence of treatment according to the guidelines is responsible for shorter survival times and not necessarily due to the comorbidity per se [20,22]. In addition, individuals might need to attend regular checkups treated at relevant clinical departments in the presence of comorbidities, leading to earlier detection of the lung cancer and thereby to early treatment [11,22]. Given this conflicting evidence in the literature, there is an important need to further explore the influence of comorbid illnesses in patient survival in order to prevent suboptimal disease management and to better tailor treatment.

In this context, the objective of this study is threefold: first, to estimate lung cancer incidence based on administrative claims data that is validated with the statistics from the national cancer registry in Germany; second, to recognize the prevalence of comorbidities among lung cancer patients in relation to individual characteristics and their evolution over time; and third, to analyze the association between comorbidities and survival rates for lung cancer patients.

## Methods

### Data source

We analyzed administrative claims data from Barmer health insurance, one of the largest statutory health insurance (SHI) funds in Germany, covering close to 9 million people (11% of the national population) [23]. Access was granted by the Scientific Data Warehouse of Barmer (W-DWH) with pseudonymized identities of the insured. The study was performed in accordance with the Declaration of Helsinki and follows established principles of good practice in secondary data analysis [24]. The dataset encompassed individual level information from inpatient and outpatient (in both hospitals and ambulatory services) records for the 15-year period from 2005 to 2019. It includes information on individual characteristics, such as sex, year of birth, place of residence, affiliation date and disenrollment date and their respective reasons. Outpatient services were registered as hospital outpatient if offered directly in a hospital and as ambulatory if offered elsewhere. The reporting system employed the German Modification for the 10^th^ revision of The International Statistical Classification of Diseases and Related Health Problems (ICD-10-GM) to list patient diagnoses. The documentation of the data source was structured in year-quarters, therefore the analysis was quarterly based.

### Patient identification

We identified incident cases on a year-by-year basis. In accordance to McGuire et al. [25] and Schwarzkopf et al. [26], patient identification was initiated by considering all individuals with a lung cancer diagnosis, classified with the ICD-10-GM code C33 or C34, in a particular year. To filter out cases in which were uncertain of diagnosis, we included only individuals with either one inpatient primary diagnosis, or two hospital outpatient or ambulatory confirmed diagnoses in consecutive quarters. Additionally, we excluded patients with prior lung cancer diagnosis or treatment in the previous two years, with the purpose of capturing only incident cases. We also omitted cases involving individuals with at least one inpatient diagnosis or one confirmed hospital or ambulatory outpatient diagnosis in the previous two years. Only individuals with Barmer insurance coverage within at least 24 months before and 6 months after the date of diagnosis (unless death occurred before) were included, to ensure sufficient records within the insurance fund. The diagnosis date for an individual was set as the earliest possible date among the first inpatient admission and the first outpatient visit to a hospital or ambulatory service. Moreover, individuals with inconsistent gender information were additionally excluded. In case of multiple places of residence, the location with the longest time in residence was chosen as the actual place of residence. Individuals younger than 18 years of age were excluded as well, in order to reduce the effect of outliers. And lastly, those insured by the DBKK, a health insurance integrated to Barmer in 2017, were excluded, as their records were incomplete for the time frame. The final panel obtained covers the period from 2007 until 2018.

### Comorbidity identification

Following guidance in Edwards et al. [27] and Morishima et al. [28], comorbidities in lung cancer patients were identified by retrieving primary and secondary inpatient diagnoses one year before and three months after the lung cancer diagnosis date. Furthermore, we employed the categories listed in the Charlson Comorbidity Index (CCI), a validated and widely used index, to classify the comorbidities into 17 groups [11,12,17,20]. We used an algorithm developed in Quan et al. [29] and Quan et al. [30] for the grouping with their respective ICD-10-GM codes. In the classification process, we excluded two comorbidity groups, namely malignancy and metastatic carcinoma, as they could be the result of lung cancer itself. Single comorbidity diagnoses as well as the number of comorbidities were employed to assess the impact of comorbid illnesses on lung cancer survival.

### Statistical analysis

Lung cancer incidence was estimated after relating the identified incident cases with the entire Barmer population. Comorbidity prevalence was calculated for each comorbidity group as the percentage of lung cancer patients diagnosed with that particular comorbidity. Survival rates were obtained for three and six months after the diagnosis date, as well as for one, three and five years after. Incidence, comorbidity prevalence and survival rates were retrieved for each year and also by sex, age at diagnosis and place of residence. Time trends in these metrics were recognized by observing three year averages across the period of analysis. If estimated averages were consistently (i.e., without exception) increasing throughout the sample, the trend was then labeled as increasing, likewise for decreasing averages. Kaplan Meier curves were estimated by censoring the sample at December 31, 2019 and excluding the patient population that terminated their insurance affiliation for reasons different than death. Confidence intervals (CI) at the 95% levels were built around the curves in order to compare surviving probabilities across subpopulations. The analysis were performed using R software, version 3.6.0.

## Results

### Lung cancer incidence

The number of new lung cancer diagnoses identified was 70,698 during the period of analysis. Table 1 presents this figure by year of diagnosis, as well as percentages relative to new diagnosis within each year and stratified by sex, age at diagnosis, and place of residence. Incident cases vary between 5,108 and 6,504 per year. It is important to highlight that, in relation to the total population of Germany, persons insured through Barmer are more frequently women, belong to older age groups, and reside more often in the state of North Rhine-Westphalia and are less often residing in the states of Baden-Wurttemberg and Bavaria. Incidence numbers are displayed in Table 2, while the same in age-standardized form appear in Appendix 1. These incidence figures were calculated with a population base without any age restriction. The latter suggests that age-standardized incidence for our sample is similar to that reported by the German Centre for Cancer Registry Data for the whole of Germany [2]. Figures for women are nearly identical; however, those for men are on average eight points below national statistics, although the gap seems to be closing in recent years.

**Table 1:** Lung cancer incident cases as percentage of total cases by sex, age group, place of residence and year of diagnosis

	2007	2008	2009	2010	2011	2012	2013	2014	2015	2016	2017	2018
Total cases	5,108	5,247	5,496	5,681	5,664	5,721	6,047	6,209	6,288	6,406	6,504	6,327
Sex												
Men	56.0	56.0	55.2	55.1	55.3	53.1	54.5	53.0	53.0	51.9	51.1	49.7
Women	44.0	44.0	44.8	44.9	44.7	46.9	45.5	47.0	47.0	48.1	48.9	50.3
Age group												
18–35	0.3	0.2	0.2	0.3	0.4	0.3	0.3	0.2	0.3	0.2	0.3	0.3
35–50	6.0	5.5	5.5	5.1	4.9	4.5	4.2	4.2	3.5	2.9	3.0	2.8
50–65	32.1	33.1	31.4	30.4	29.6	31.6	31.2	31.0	31.3	29.9	30.1	28.1
65–80	48.1	47.9	48.4	49.8	50.5	49.8	50.2	50.7	52.1	52.5	52.0	52.7
80+	13.5	13.3	14.5	14.4	14.6	13.8	14.2	14.0	12.8	14.6	14.5	16.1
Place of residence												
Baden-Wurttem.	7.0	6.9	7.2	7.5	6.7	7.1	7.3	7.0	6.6	7.0	7.1	7.1
Bavaria	10.3	10.5	9.5	10.4	10.9	9.8	9.7	9.8	10.3	9.4	10.1	10.0
Berlin	6.1	5.8	5.2	6.0	5.6	5.5	6.1	6.1	5.9	5.7	5.7	5.7
Brandenburg	3.2	3.5	3.7	3.7	3.8	4.4	4.4	4.6	4.4	4.4	4.6	4.4
Bremen	0.5	0.5	0.6	0.4	0.7	0.4	0.5	0.5	0.4	0.5	0.5	0.6
Hamburg	2.5	2.3	2.4	2.1	2.2	2.4	2.7	2.7	2.7	2.8	3.0	2.6
Hesse	8.1	7.8	7.2	7.9	7.0	7.5	8.1	8.0	7.4	7.9	7.1	8.0
Mecklenburg-Vor.	1.9	2.1	1.9	2.4	2.3	2.0	2.4	2.2	2.3	2.4	2.6	2.1
Lower Scaxony	8.4	8.7	9.0	8.6	8.4	8.2	8.1	8.8	8.7	9.2	8.8	8.5
North rhine-West.	31.3	30.8	31.0	30.0	30.0	30.6	29.1	29.9	30.6	29.5	29.3	30.3
Rhineland-Palat.	5.6	5.6	5.7	5.4	5.8	6.2	5.7	4.8	5.5	5.8	5.8	5.6
Saarland	1.5	1.8	1.5	1.5	1.4	1.5	1.7	1.7	1.4	1.3	1.8	1.7
Saxony	4.1	3.5	4.1	4.2	4.3	4.4	3.8	3.7	3.6	3.2	3.5	3.5
Saxony-Anhalt	2.8	2.7	3.1	2.8	3.0	2.7	3.1	3.2	2.7	2.8	2.7	2.9
Schleswig-Hols.	4.5	5.3	5.1	4.8	5.0	4.5	4.7	5.1	4.9	5.4	5.0	4.8
Thuringia	2.1	2.2	2.5	2.3	2.8	2.8	2.5	2.0	2.5	2.7	2.4	2.4

**Table 2:** Lung cancer incidence per 100,000 individuals by sex, age group, place of residence and year of diagnosis

	2007	2008	2009	2010	2011	2012	2013	2014	2015	2016	2017	2018
Total (+)	60.3	62.0	65.0	67.0	66.2	66.4	70.3	72.6	74.4	77.2	79.3	78.4
Sex												
Men (+)	82.7	84.7	87.3	89.4	88.4	84.8	91.9	91.8	93.7	94.9	95.7	91.8
Women (+)	57.1	58.9	61.0	62.9	62.5	60.4	65.8	66.3	68.1	69.3	70.4	67.7
Age group												
18–35	0.9	0.8	0.6	1.0	1.3	1.0	1.0	0.6	1.2	0.7	1.5	1.3
35–50 (−)	16.4	15.8	17.0	16.8	16.1	15.4	15.6	16.4	14.6	12.7	13.7	12.9
50–65	93.6	98.6	97.5	94.9	89.2	93.9	96.5	97.8	100.1	98.1	100.7	92.4
65–80 (+)	171.3	170.5	176.4	187.7	188.0	183.0	191.8	197.8	206.2	213.6	216.5	215.9
80+	156.5	152.8	170.3	169.8	169.1	158.2	170.5	164.8	145.4	159.3	152.5	154.9
Place of residence												
Baden-Wurttem. (+)	48.0	48.4	52.6	56.1	49.8	53.2	57.6	57.4	55.1	60.6	63.2	62.9
Bavaria (+)	50.3	52.4	50.1	56.7	59.1	53.0	56.1	58.2	62.0	58.3	64.4	62.7
Berlin (+)	77.1	74.8	70.5	84.5	78.3	75.9	89.3	92.1	92.0	92.1	93.7	92.7
Brandenburg (+)	44.8	50.3	55.8	56.7	57.1	64.3	68.2	72.5	71.0	73.5	78.3	73.2
Bremen	74.9	75.5	91.9	67.5	103.0	65.6	82.6	88.9	72.7	99.9	90.0	104.0
Hamburg	72.3	68.7	76.9	69.2	70.6	76.1	94.6	95.4	99.4	104.7	115.3	98.9
Hesse (+)	58.5	57.3	55.5	62.7	55.0	58.8	66.8	68.0	64.3	69.9	64.9	71.0
Mecklenburg-Vor. (+)	41.1	44.6	43.9	54.3	51.5	44.9	55.5	52.1	53.5	58.9	62.7	49.8
Lower saxony (+)	58.9	62.2	68.0	66.0	64.2	63.0	65.6	73.4	74.4	82.3	82.2	80.4
North rhine-West. (+)	76.0	77.4	81.8	81.8	81.3	83.6	84.4	89.7	93.7	93.1	94.4	96.0
Rhineland-Palat. (+)	69.5	72.3	76.6	75.4	80.6	85.7	84.1	73.2	85.1	91.7	94.4	88.5
Saarland	57.1	70.6	61.9	63.2	59.3	61.2	73.9	76.3	66.2	61.3	87.8	79.6
Saxony	50.3	44.5	54.4	57.2	58.0	59.6	55.7	55.6	59.2	56.7	66.6	66.5
Saxony-Anhalt (+)	47.6	48.3	58.1	53.3	55.8	50.8	61.1	65.8	59.0	65.1	64.6	69.5
Schleswig-Hols. (+)	131.6	159.7	162.3	155.7	160.6	146.6	162.5	182.2	179.0	204.1	196.0	183.8
Thuringia	45.6	48.9	59.9	55.6	65.8	66.5	62.5	51.9	70.0	81.1	76.2	76.7

Note: If the three year average is always higher (lower) than the three year average of the period immediately before, the trend is labeled as increased (decreasing). An increasing trend in incidence is denoted by (+), a decreasing trend by (−).

Table 2 reveals an overall increasing trend in incidence throughout the entire time period, starting at 60.3 cases per 100,000 individuals in 2007 and ending at 78.4 cases per 100,000 individuals in 2018. This trend was observed in both men and women, although incidence in men is close to 50% higher than incidence in women. Incidence is highest in the age group 65–80, and it is also the only age group which exhibited an increasing trend, growing from 171.3 cases per 100,000 individuals to 215.9 cases per 100,000 individuals. In contrast, incidence steadily decreased in the age group 35–50 over the whole period. The states of Bremen, Hamburg, Saarland, Saxony and Thuringia presented stable incidence figures. All other states showed an increasing trend. Incidence was particularly high in Schleswig-Holstein with around 100 cases per 100,000 above the sample average, and particularly low in Mecklenburg-Vorpommern with almost 20 cases per 100,000 below the sample average.

### Comorbidity prevalence

Comorbidity prevalence statistics can be found in Table 3. It displays the percentage of lung cancer patients with a particular comorbidity diagnosis, as well as with a number of comorbidities. As shown, close to 30% of lung cancer patients were not affected by any comorbidities, while 50% were by one or two comorbidities, 15% by three or four, and less than 5% by five or more. It is also noteworthy that the percentages of lung cancer patients without comorbidities had a downward trajectory over the period of analysis, while those with three and more an upward trend. Chronic obstructive pulmonary disease (COPD) was by far the most common comorbidity, diagnosed in more than a third of lung cancer patients. It was followed by peripheral vascular disease (PVD), diabetes without chronic complications, congestive heart failure (CHF) and renal disease, affecting between 10% and 20% of lung cancer patients on average and presenting an increasing trend over time. Peptic ulcer was the only comorbidity with a decreasing trend within the timeframe analyzed.

**Table 3:** Comorbidity prevalence as percentage of lung cancer patients by comorbidity type, number of comorbidities and year of diagnosis

	2007	2008	2009	2010	2011	2012	2013	2014	2015	2016	2017	2018
Comorbidity												
Myocardial infarction	6.6	5.6	5.6	6.2	6.1	5.5	6.2	6.9	6.6	6.8	7.0	6.3
CHF (+)	15.6	15.3	15.4	15.2	15.8	15.5	17.6	17.5	16.6	17.7	17.6	17.5
PVD (+)	13.4	14.5	16.3	17.1	17.5	19.0	20.2	20.1	20.6	21.3	21.0	20.8
CEVD (+)	9.0	8.3	8.0	8.6	8.7	8.9	9.2	10.0	9.1	10.6	10.8	10.3
Dementia	3.3	3.3	3.1	3.5	3.1	3.3	2.9	3.4	2.9	2.8	2.9	3.3
COPD (+)	35.1	34.1	34.7	35.9	37.0	36.4	35.6	38.3	37.4	38.8	38.1	37.9
Rheumatoid disease (+)	1.6	1.8	1.8	1.9	1.8	2.1	2.0	2.8	2.3	2.5	2.5	2.5
Peptic ulcer (−)	3.1	3.1	3.0	2.8	2.8	2.9	2.6	2.4	2.4	2.5	2.6	2.2
Mild liver disease	4.7	4.5	4.4	4.6	5.0	4.9	4.4	4.6	5.1	5.2	5.7	6.1
Diab. without chron. compl.	15.8	15.8	16.0	16.1	17.4	17.8	19.0	18.5	18.0	17.5	18.3	17.9
Diab. with chron. comp.	4.2	4.0	4.1	3.9	4.1	4.2	4.3	4.7	4.5	4.5	5.0	4.7
Hemiplegia or paraplegia	5.3	5.1	4.7	5.4	5.4	5.5	5.3	5.3	5.3	5.2	5.6	5.6
Renal disease (+)	11.5	11.4	12.4	12.9	14.1	14.8	16.1	15.8	15.8	16.7	16.3	16.6
Moderate or severe liver dis.	0.6	0.7	0.5	0.5	0.7	0.6	0.6	0.8	0.8	0.8	0.6	0.8
AIDS	0.1	0.1	0.1	0.0	0.1	0.1	0.1	0.1	0.1	0.2	0.1	0.0
Number of comorbidities												
0 (−)	34.4	35.6	33.8	33.1	31.6	30.9	29.7	27.8	29.6	27.9	28.9	27.2
1 or 2	49.4	48.6	50.3	49.5	50.2	50.9	50.7	52.2	50.3	51.1	49.6	52.3
3 or 4 (+)	13.8	13.3	13.5	14.6	14.8	14.7	16.4	16.4	16.5	17.2	17.5	17.0
5 or more (+)	2.3	2.5	2.4	2.7	3.4	3.5	3.2	3.6	3.5	3.8	4.0	3.5

Note: Comorbidities are classified with the categories listed in the Charlson Comorbidity Index (CCI). COPD stands for chronic obstructive pulmonary disease, PVD for peripheral vascular disease, CHF for congestive heart failure and CEVD for cerebrovascular disease. If the three year average is always higher (lower) than the three year average of the period immediately before, the trend is labeled as increased (decreasing). An increasing trend in incidence is denoted by (+), a decreasing trend by (−).

Table 4 presents comorbidity prevalence numbers by sex and age group. It suggests that men and older individuals have relatively more comorbidities than other lung cancer patients. The five most prevalent comorbidities, previously mentioned, were the same in both men and women; however, these affect men on average 5% more often than women. Moreover, COPD was prevalent in more than 10% of the lung cancer patients in the youngest age group. This 10% threshold was also reached by PVD and diabetes without chronic complications in the 35–50 age group, as well as by CHF, renal disease and cerebrovascular disease (CEVD) in the 65–80 age group, and finally by dementia in the 80+ age group.

**Table 4:** Comorbidity prevalence as percentage of lung cancer incident cases in 2008–2018 by sex and age group

	Men	Women	18–35	35–50	50–65	65–80	80+
Comorbidity							
Myocardial infarction	8.6	3.7	0.0	1.6	4.1	7.5	8.3
CHF	19.1	13.5	2.2	4.4	9.5	18.4	28.9
PVD	21.4	15.5	7.1	10.8	16.3	21.2	17.4
CEVD	10.5	8.0	2.2	2.7	6.4	10.7	13.1
Dementia	3.2	3.1	0.0	0.0	0.6	3.0	10.1
COPD	38.7	34.4	16.9	24.6	36.2	39.8	30.9
Rheumatoid disease	1.4	3.0	0.6	1.2	2.1	2.3	2.0
Peptic ulcer	3.0	2.3	1.6	1.6	2.4	2.9	3.1
Mild liver disease	5.3	4.6	1.6	5.1	5.9	4.9	3.2
Diab. without chron. compl.	20.9	13.4	3.3	4.8	13.1	20.6	19.4
Diab. with chron. compl.	5.7	2.8	0.0	0.6	2.5	5.4	6.1
Hemiplegia or paraplegia	5.5	5.1	2.2	3.8	5.6	5.4	4.9
Renal disease	17.2	11.7	2.2	3.0	6.8	16.7	28.1
Moderate or severe liver dis.	0.9	0.4	0.0	0.6	0.8	0.7	0.4
AIDS	0.1	0.0	0.5	0.4	0.2	0.0	0.0
Number of comorbidities							
0	26.2	35.8	68.3	54.8	36.4	26.6	24.8
1 or 2	50.9	50.0	30.6	41.5	52.2	51.1	47.7
3 or 4	18.8	12.0	1.1	3.2	10.0	18.2	22.3
5 or more	4.2	2.2	0.0	0.4	1.5	4.0	5.2

Note: Comorbidities are classified with the categories listed in the Charlson Comorbidity Index (CCI). COPD stands for chronic obstructive pulmonary disease, PVD for peripheral vascular disease, CHF for congestive heart failure and CEVD for cerebrovascular disease.

### Lung cancer survival

The survival rate is presented in Table 5 for different survival times. Most recent estimations obtained in year 2018 were 19.7% (five-year survival rate), 27.8% (three-year), 51.2% (one-year), 67.4% (three-month) and 80.8% (one-month). In addition, survival rates improved across time for the longer time windows (five-year, three-year and one-year) while they remained stable for the shorter time windows (six-month and three-month). Survival was consistently higher for women than men, with differences under 10 percentage points. For men, an upward trend is observed in the five-year and three-year survival rates. In women, an upward trend was observed in the three-year survival rate, while a downward trend in the five-year survival. Most of these trends were also identified by the German Centre for Cancer Registry Data for the whole of Germany, as seen in Appendix 2 [2]. Nevertheless, our survival numbers are on average 5% higher, with differences being less accented in men and for the five-year mark. Figures provided by the Global Surveillance of Cancer Survival Programme (CONCORD-3) are more relatable. As shown in Appendix 3, they estimated a five-year age-standardized survival rate of 18.3% in 2010 for Germany, which was 3% lower than our rate for the same year [31]. Furthermore, the survival rate was lower in the older age groups (Table 5), regardless of the time window analyzed. Differences were, however, more pronounced for longer time frames. For example, the difference between the youngest and the oldest age group is nearly 60 percentage points for the five-year survival, and almost 30 percentage for the three-month survival. Improvement in survival rates were observed more often, but not exclusively, in the 50–65 and 65–80 ages groups during the sample period. Declines were seen only for the 18–35 and 80+ age groups in the five-year survival.

**Table 5:** Survival probability of lung cancer incident cases by survival time, sex, age group and year of diagnosis

	2008	2009	2010	2011	2012	2013	2014	2015	2016	2017	2018
5-year survival											
Total (+)	19.0	18.6	18.9	19.7	18.8	19.8	19.7	–	–	–	–
Sex											
Men (+)	14.9	15.6	15.2	16.9	15.4	16.7	17.9	–	–	–	–
Women (−)	24.2	22.5	23.4	23.2	22.9	23.4	21.9	–	–	–	–
Age group											
18–35 (−)	71.4	75.0	88.9	62.5	52.4	68.8	56.2	–	–	–	–
35–50 (+)	28.3	27.7	29.6	28.9	30.9	32.8	38.6	–	–	–	–
50–65	22.0	22.4	22.9	24.6	22.4	24.4	22.2	–	–	–	–
65–80 (+)	18.5	18.0	17.9	19.2	18.5	18.6	20.1	–	–	–	–
80+ (−)	8.3	6.6	8.5	7.0	7.2	8.4	6.6	–	–	–	–
3-year survival											
Total (+)	24.4	24.7	25.3	26.1	25.4	26.7	26.2	26.9	27.8	–	–
Sex											
Men (+)	20.5	21.2	21.1	22.8	22.1	23.7	24.0	23.5	23.9	–	–
Women (+)	29.3	29.2	30.5	30.2	29.5	30.1	28.9	30.8	32.1	–	–
Age group											
18–35	71.4	75.0	88.9	62.5	57.1	68.8	56.2	80.0	66.7	–	–
5-50 (+)	33.2	33.2	34.9	34.0	37.5	38.2	41.7	45.0	47.5	–	–
50–65	27.2	28.5	29.6	30.2	28.3	31.0	28.9	29.3	31.3	–	–
65–80 (+)	24.3	24.7	24.4	26.1	25.7	26.0	26.9	27.5	27.5	–	–
80+	12.8	10.8	14.8	14.1	13.8	15.2	12.7	13.9	13.9	–	–
1-year survival											
Total (+)	48.5	48.6	48.8	49.9	49.3	50.5	49.5	50.7	51.4	50.6	51.2
Sex											
Men	45.0	45.2	45.0	45.7	46.0	47.5	47.1	47.6	48.1	46.1	48.1
Women	53.0	52.7	53.6	55.1	53.4	53.9	52.3	54.3	55.1	55.5	54.4
Age group											
18–35	92.9	83.3	88.9	93.8	85.7	75.0	75.0	80.0	83.3	90.0	72.7
35–50	60.6	60.2	54.5	59.5	64.7	62.2	62.6	69.0	67.7	59.1	69.9
50–65 (+)	51.8	53.5	55.0	53.9	55.2	55.9	55.1	55.0	56.1	57.0	56.6
65–80 (+)	49.4	49.2	48.1	50.6	49.2	50.5	49.5	50.8	51.4	51.0	51.0
80+ (+)	31.1	28.6	35.2	34.8	31.5	33.5	32.5	35.1	35.0	34.2	36.1
6-month survival											
Total	67.5	67.0	67.3	68.3	67.2	68.1	67.4	68.1	68.1	68.4	67.4
Sex											
Men	65.7	64.5	64.5	65.2	65.1	65.7	66.2	66.8	65.7	64.7	65.2
Women	69.9	70.3	70.8	72.1	69.8	70.8	68.8	69.5	70.8	72.4	69.7
Age group											
18–35	100.0	100.0	88.9	93.8	95.2	93.8	87.5	100.0	88.9	100.0	90.9
35–50	77.5	78.9	76.4	80.8	79.3	80.7	80.7	83.7	82.5	79.0	82.1
50–65 (+)	73.0	72.9	72.4	73.9	73.3	74.1	73.6	74.2	73.6	76.1	72.6
65–80	67.5	66.9	67.8	67.9	67.6	67.9	67.2	67.7	68.2	68.2	67.4
80+	49.3	47.4	51.1	53.2	48.7	50.3	50.3	51.1	49.9	50.9	52.8
3-month survival											
Total	0.81	0.81	0.81	0.81	0.81	0.81	0.81	0.81	0.82	0.81	0.81
Sex											
Men	0.80	0.79	0.79	0.79	0.80	0.80	0.80	0.80	0.81	0.79	0.80
Women	0.82	0.84	0.84	0.84	0.82	0.83	0.82	0.82	0.84	0.84	0.82
Age group											
18–35	1.00	1.00	1.00	1.00	1.00	0.94	1.00	1.00	0.94	1.00	1.00
35–50	0.87	0.89	0.88	0.91	0.91	0.92	0.89	0.93	0.92	0.89	0.92
50–65 (+)	0.85	0.86	0.84	0.86	0.86	0.85	0.85	0.85	0.87	0.87	0.85
65–80	0.82	0.81	0.81	0.81	0.81	0.81	0.81	0.81	0.82	0.81	0.80
80+ (+)	0.65	0.67	0.71	0.70	0.67	0.70	0.69	0.70	0.70	0.68	0.72

Note: If the three year average is always higher (lower) than the three year average of the period immediately before, the trend is labeled as increased (decreasing). An increasing trend in survival is denoted by (+), a decreasing trend by (−).

### Lung cancer survival and comorbidity

**Figure 1:**
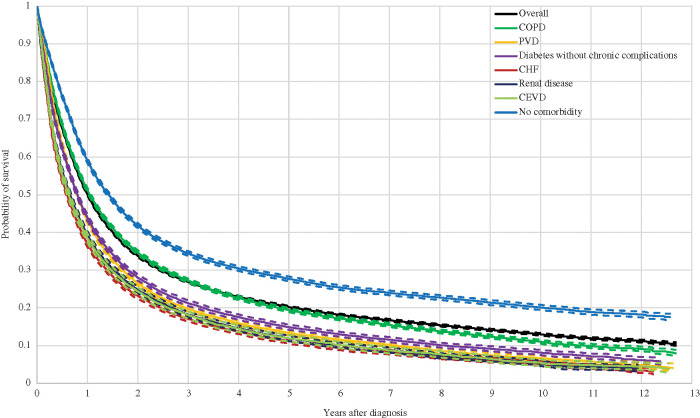
Kaplan Meier Curves and 95% Confidence Interval for Lung Cancer Patients with Most Common Comorbidities Note: Comorbidities are classified with the categories listed in the Charlson Comorbidity Index (CCI). COPD stands for chronic obstructive pulmonary disease, PVD for peripheral vascular disease, CHF for congestive heart failure and CEVD for cerebrovascular disease.

Fig. 1 depicts the Kaplan-Meier curves for all lung cancer patients identified in the sample, as well as for those diagnosed with the most comorbidities or affecting at least 10% of lung cancer patients. As observed, except for COPD, a diagnosis from any of the major comorbidities is associated with a significantly lower probability of survival. Relative to overall survival, the largest drops were observed for CHF (13%, 10%, 9% and 8% less in one-year, three-year, five-year and ten-year survival probabilities, respectively), CEVD (12%, 9%, 8% and 8% less) and renal disease (11%, 9%, 8% and 8% less). Smaller drops were found for PVD (7%, 7%, 7% and 6% less) and diabetes without chronic complications (6%, 6%, 5% and 5% less). The probability of survival associated with COPD was significantly lower than the overall survival only after six years from the diagnosis and this difference was never larger than 2%. On the other hand, patients not reporting any comorbidity were associated with higher survival probabilities (8%, 8%, 7% and 7% more).

**Figure 2:**
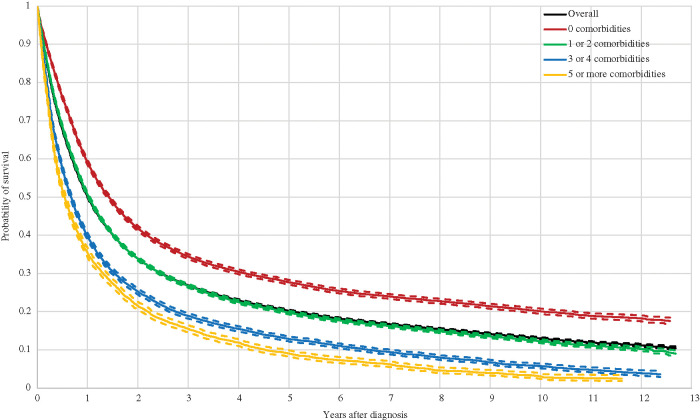
Kaplan Meier Curves and 95% Confidence Interval for Lung Cancer Patients by Number of Comorbidities Note: Comorbidities are classified with the categories listed in the Charlson Comorbidity Index (CCI).

Moreover, Kaplan-Meier curves by the number of comorbidities are shown in Fig. 2. As shown, the curves are nearly identical for the overall sample and for the group with one or two comorbidities. Even though the mean survival probability was slightly lower for the latter, it is never significantly different than that of the overall sample at any point after the diagnosis. In contrast, the toll for the remaining groups was large: the probability of survival was 10%, 8%, 8% and 7% lower for the one-year, three-year, five-year and ten-year time windows respectively for the group with three or four comorbidities, and 15%, 11%, 11% and 10% lower for the group with five comorbidities or more.

## Discussion

The present study identified 70,698 lung cancer incident cases covering all of Germany and across a 12-year period between 2007 and 2018, making it the largest lung cancer patient sample—to which we are aware—in Germany. We validated the representativeness of the sample by comparing our obtained age-standardized incidence figures with those from the German Centre for Cancer Registry Data, which is responsible for pooling and assessing every reported cancer case it receives from the population based cancer registries in each German federal state. Our incidence for women was nearly identical to national statistics over the 12 years analyzed; though, our incidence for men was higher [2]. This might be partially explained by the socioeconomic background of patients insured with Barmer [32]. While Barmer has traditionally been a health insurance fund for while-collar workers, lung cancer appears to be more common among low income individuals in Germany, due to higher smoking rates [33,34]. Moreover, the overall increasing trend in lung cancer age-standardized incidence in Germany for the 2007–2018 period is consistent with numbers for the whole of Germany from the German Centre for Cancer Registry Data [2].

Comorbidity prevalence in our sample was in line to that found in Islam et al. [12]. However, the analyses by Iachina et al. [11] and Nilsson et al. [20] identified a smaller proportion of lung cancer patients with comorbidities, at 53% and 44% respectively. This outcome might be explained by both studies focusing on non-small cell lung cancer (NSCLC) patients, for which a smoking history is usually less common [35]. In addition, these studies retrieved their comorbidity profiles from patient registries, which tend to report diagnostic profiles in less detail compared to administrative claims or medical records, for incentive reasons [36,37]. This is also the case in Seigneurin et al. [21], in which 56% of lung cancer patients bear at least one comorbidity. On the other hand, Tammemagi et al. [16] and Grose et al. [5] reported larger numbers of lung cancer patients with comorbidities, 88% and 87%, respectively. However, they included a longer comorbidity list compared to that of the CCI. Comorbidity prevalence found in Wang et al. [17] and Feng et al. [38] was higher as well at 81% and 78% respectively, most likely due to the samples being restricted to older individuals who tend to bear more comorbidities.

The most prevalent concomitant diseases in our study were similar to those in the Annual Report to the Nation on the Status of Cancer, based on a sample of more than 160,000 lung cancer patients in the US [27]. The only exception was for the prevalence of renal disease, for which we estimated a considerably larger percentage, presumably because the latter identified only cases of chronic renal disease. Likewise, at least three of the five most prevalent comorbidities in Islam et al. [12], Grose et al. [5], Murawski et al. [6], and Tammemagi et al. [16] coincided with the five most frequent comorbidities in our sample. Nevertheless, the proportion of lung cancer patients with COPD is lower in Islam et al. [12] and Murawski et al. [6] compared to our results, most likely due to sample differences. Relative to our sample, patients are on average older in Islam et al. [12] and predominantly male in Murawski et al. [6], for which COPD tends to be more prevalent [39]. Other comorbidities that were highly prevalent in previous research were either illness symptoms or severity indicators that are not covered by the CCI.

With respect to survival, time trends for the short- and long-term, as well as divergence across sex, found in this study, were also identified by the German Centre for Cancer Registry Data for the whole of Germany [40]. Nevertheless, our survival probabilities were on average higher, with less discrepancy for men and the overall five-year mark. These differences could be masking disparities between the age structure of our sample and German population. In this regard, relatable age-standardized survival rates are provided by the Global Surveillance of Cancer Survival Programme (CONCORD-3), although our estimation is still slightly higher.

A greater probability of survival among lung cancer patients without comorbidities was also observed in Islam et al. [12], Tammemagi et al. [16], Kravchenko et al. [13], Welch et al. [22] and García-Pardo et al. [10]. However, our estimated survival rates cannot be directly compared, given that these studies controlled for other patient characteristics. In contrast, Seigneurin et al. [21] and Murawski et al. [6] found that lung cancer patients were not necessarily better off in terms of survival when comorbidity free, although significant differences did exist by histology in Seigneurin et al. [21] and by treatment regime in Murawski et al. [6]. Nonetheless, Murawski et al. [6] did not control for cancer stage and histology, which may explain this non-negative effect. Seigneurin et al. [21] did control for these and nevertheless obtained a non-significant effect of comorbidities on survival among NSCLC patients. Furthermore, the relatively small or even non-significant association between survival and COPD, the most frequent comorbidity, was supported by Islam et al. [12] and Tammemagi et al. [16]. Patients with COPD were more often diagnosed at earlier lung cancer stages, most likely as a consequence of close monitoring by a physician or treatment at relevant clinical departments that thereby have the potential opportunity for earlier detection [11]. Except for PVD, Islam et al. [12] and Tammemagi et al. [16] also found a negative association between survival and the remaining most important five comorbidities (PVD, diabetes without complications, CHF, renal disease and CEVD). The lower survival in lung cancer patients with CHF might be caused from lower likelihoods of undergoing surgery and chemotherapy, in particular in earlier stages where the presence or absence of CHF is crucial in the treatment choice [9,16]. A similar conclusion was provided by Welch et al. [22]. In contrast, Kravchenko et al. [13] associated lower lung cancer survival with increasing operative mortality among patients with CHF. As for lung cancer patients with renal disease, poorer survival probabilities could result from modified doses or complete discontinuation of chemotherapy at later stages, which is often platinum based and therefore not recommendable for those with kidney disease [12]. For PVD, the effect was not significant in the results of Islam et al. [12] and even positive in Tammemagi et al. [16]. This could suggest that the probability of survival in lung cancer patients with PVD was essentially explained by other treatment and individual effects rather than the comorbidity itself.

Overall, this study provides a detailed picture on the comorbidity profile of lung cancer patients in Germany with a representative sample covering all sixteen federal states and a period of time longer than a decade. It is necessary to supply figures in this matter, as the German Centre for Cancer Registry Data does not collect information on comorbidities, a crucial element in understanding lung cancer survival. In this respect, we have delivered evidence on its association with most common comorbidities, and thereby a clearer characterization of lung cancer prognosis. This could facilitate improvement in treatment selection and strategies that enhance survival and quality of life in lung cancer patients.

### Limitations

The main limitation of our analysis was the inability to control for individual characteristics that might influence lung cancer patient survival. This is because administrative data does not include information on, for instance, tumor stage and histology. We cannot, therefore, disentangle the effect of comorbidities on survival rates by means of multivariate regression methods. Tumor stage and histology have been shown to explain most variation in lung cancer survival, and by excluding them, an econometric analysis would inevitably suffer from omitted variable biases [4,16,21,41]. For this reason, the reader should interpret our results as associations rather than causation.

## Conclusions

The present study reveals the epidemiological picture of lung cancer incidence and comorbidity prevalence, as well as provides evidence on the relationship between survival and comorbidities among lung cancer patients, based on administrative claims data from a health insurance company in Germany. The sample was large and the patient identification strategy delivered incidence and survival figures that were comparable to official statistics for lung cancer patients in Germany. Comorbidities are prevalent in around 70% of lung cancer patients and usually associated with lower probability of survival. The largest impact on survival from common comorbidities is observed in lung cancer patients with CHF, CEVD or renal disease, and a more moderate impact in those with PVD or diabetes without chronic complications. Further research for Germany is needed to disentangle the effect of comorbidities on survival from other patient characteristics such as cancer stage and histology.

## Data Availability

This study is supported by Barmer, with whom the Division of Health Economics has a Data Use and Transfer Agreement. Personal data of the beneficiaries were pseudonymized through Barmer before data sharing. Personal identifiers were masked or deleted prior to receiving the data. Quasi-identifiers were generalized (year of birth only, deletion of the last two digit of the zip code, etc.) The processing and analysis of sensitive data took place by remotely accessing the servers at the Scientific Data Warehouse of Barmer under special data protection conditions. The Data Use and Transfer Agreement does not contemplate data distribution to third parties and it is therefore not available.

## Appendix

**Appendix 1:** Age standardized lung cancer incidence by year of diagnosis

	2007	2008	2009	2010	2011	2012	2013	2014	2015	2016	2017	2018
Barmer sample												
Women	26.0	26.6	27.4	28.0	27.1	28.3	28.3	29.8	30.8	31.0	32.5	31.7
Men	53.2	53.7	53.5	54.0	52.5	50.6	53.7	52.7	53.3	52.6	52.5	49.1
Germany												
Women	25.9	26.4	26.6	27.4	28.4	29.1	29.8	30.4	31.3	31.6	31.7	31.5
Men	65.8	65.2	64.7	63.3	62.9	61.5	61.1	59.8	59.6	58.1	58.2	55.3

Note: Age standardized incidence is calculated for the sample with the European Standard Population 1976. Figures for Germany are retrieved from [2].

**Appendix 2:** Survival probability of lung cancer incident cases by year of diagnosis

	2007–2008	2009–2010	2011–2012	2013–2014	2015–2016	2017–2018
Barmer sample						
5-year survival						
Women	23	23	23	22	–	–
Men	15	16	16	18	–	–
3-year survival						
Women	29	30	30	30	32	–
Men	21	22	23	24	24	–
1-year survival						
Women	53	54	54	53	55	55
Men	45	45	47	47	47	47
Germany						
5-year survival						
Women	18	19	18	19	19	20
Men	12	13	13	13	13	15
3-year survival						
Women	23	24	24	25	25	27
Men	17	19	18	18	18	21
1-year survival						
Women	48	48	48	49	48	49
Men	41	43	41	41	40	43

Note: Figures for Germany are retrieved from [2].

**Appendix 3:** Age standardized survival probability of lung cancer incident cases by year of diagnosis.

	2007	2008	2009	2010	2011	2012	2013
Barmer sample							
5-year survival	20.8	19.8	20.7	21.3	20.8	21.9	22.6
CONCORD-3							
5-year survival				18.3			

Note: Age standardized survival probability is calculated for the sample with the European Standard Population 1976. Figures for Germany are retrieved from [31].
